# How does perceived respect affect innovative behavior? The role of thriving at work and spiritual leadership

**DOI:** 10.3389/fpsyg.2022.978042

**Published:** 2022-10-06

**Authors:** Li Zhao, Pingqing Liu, Fan Zhang, Shuang Xu, Yuanyuan Liu

**Affiliations:** ^1^School of Management and Economics, Beijing Institute of Technology, Beijing, China; ^2^School of Geographical Science and Tourism, Nanyang Normal University, Nanyang, China; ^3^School of Economics Management, Beijing University of Agriculture, Beijing, China

**Keywords:** perceived respect, innovative behavior, thriving at work, spiritual leadership, broaden-and-build theory

## Abstract

Many enterprises use innovation to deal with the rapidly changing business environment and gain market competitiveness. How to internally motivate employees, especially the new generation of employees (e.g., employees born after 1980), to take initiative to innovate is receiving great interest from both academic and practical perspectives. Based on the broaden-and-build theory, this study presents a moderated mediation model of the impact of perceived respect on innovative behavior. SPSS and Mplus were used to analyze the data from 506 leader–employee pairs. The results show that perceived respect had a significant positive influence on innovative behavior through the effect of thriving at work, and the moderating effect of spiritual leadership was significant. When the effect of spiritual leadership was strong, the effect of perceived respect on innovative behavior through the effect of thriving at work was enhanced. This study reveals the dynamic mechanisms of improving employees’ innovative behavior, providing theoretical and practical ideas for promoting enterprises’ sustainable and innovative development.

## Introduction

In this volatile, uncertain, complex, and ambiguous era, innovation plays a critical role in improving socioeconomic prosperity (Jaiswal and Dhar, 2017) and has become increasingly important to organizational performance and survival (Anderson et al., 2014). Because employees are the direct practitioners of enterprise innovation, their innovative behavior is closely related to an enterprise’s innovative ability and performance (Shalley and Zhou, 2008). The new generation of employees (e.g., employees born after 1980) have become the backbone of the contemporary workforce (Ren et al., 2018), and their innovation is particularly important for the development of organizations. How to promote the innovative behavior of the new generation of employees has received a great deal of attention from both academic and practical perspectives (Dewangan and Godse, 2014). Therefore, the research object of this paper mainly refers to the new generation of employees. According to the characteristics of the new generation employees, we conduct targeted research on how to stimulate the employees’ innovative behavior.

The new generation of employees is well educated and concerned more about their feelings and experience at work than older employees (Zhu et al., 2015). For example, they pay more attention to justice and fairness and have a stronger desire for recognition, value, and respect (Weyland, 2011). Those who perceive respect and recognition from the organization show more positive attitudes and behaviors conducive to innovation, e.g., enhanced creativity, active absorption of new information, and acquisition of new knowledge (Amabile et al., 2005; Ng, 2016). Thus, perceived respect may be a vital factor in promoting innovative behavior. It is of great significance to investigate the influence of employees’ perceived respect on their innovative behaviors. Perceived respect is an important embodiment of employees’ spiritual needs being met. It is the employees’ inner feeling that they are recognized and encouraged by the organization (Rogers and Ashforth, 2017), which is largely consistent with Fredrickson’s concept of positive emotions. Therefore, perceived respect is an uplifting and positive emotional response to being inspired or appreciated. However, studies on the effect of perceived respect on employee behavior have mainly regarded perceived respect as an organizational environmental factor (Carmeli et al., 2013; Ng et al., 2021), ignoring its influence as an emotional factor. Internal emotional factors have a stronger and more lasting effect than external factors on promoting the new generation of employees’ inherent initiative to conduct positive behavior for the organization (Zhou et al., 2014), which is the basic driving force of employees’ innovative behavior (Bani-Melhem et al., 2018). Most research on the antecedents of employees’ innovative behavior has focused on factors at the individual level such as employees’ personality (Baer, 2010; Anderson et al., 2014; Gu et al., 2015; Liang et al., 2022) and external motivation factors such as organizational culture and supportive investment (Lin, 2011; Swart et al., 2014; Tan et al., 2021; Mustafa et al., 2022), while the influence of positive emotions on employees’ innovative behavior and the mechanism of this influence remain unaddressed. This study aims to fill this gap by exploring the internal mechanism explaining the impact of perceived respect on innovative behavior.

According to the broaden-and-build theory, positive emotion can not only broaden the scope of an individual’s attention, cognition, and action but also help build enduring personal resources (Fredrickson, 2001). Thriving at work, which can be enhanced by perceived respect, refers to employees’ positive psychological state of experiencing both vitality and learning at work (Spreitzer et al., 2005). Satisfying employees’ need to be respected could make them more optimistic, confident, and energetic, which could help strengthen their personal skills in the short term, as well as their intellectual capacity in the long term (Spreitzer and Porath, 2013; Prem et al., 2017). Sustainable innovation requires employees to be active, willing to learn, and able to put what they have learned and created into work. Accordingly, thriving at work echoes the need for sustainable innovation. Thus, we take thriving at work as the mediator in the influence mechanism of employees’ perceived respect on innovative behavior.

As innovation requires support from the work situation, especially the leadership as the representative of the organization. The development of employees’ innovative behavior is inevitably influenced by the leader (Spreitzer et al., 2005). Leadership style is an important situational variable that affects the generation and development of employees’ innovative ideas, ability, motivation, and behavior (Janssen and Van Yperen, 2004). Employees’ perception of respect is derived from how they perceive their evaluation by others in the workplace, especially leaders (Ng, 2016). Spiritual leadership, which is an important source of perceived respect, can satisfy employees’ psychological needs through intrinsic motivation, for example by setting a clear vision for employees, endowing them with faith and hope, and conveying altruistic love with care and concern (Fry, 2003). Therefore, the function of spiritual leadership is consistent with the connotations and requirements of respect. In addition, under the influence of spiritual leadership, employees may show increased willingness to maintain full energy and enthusiasm for learning and to explore innovative methods and improvement plans. Therefore, spiritual leadership could strengthen the effect of perceived respect and enhance the degree of employees’ thriving and innovative behavior from the perspective of inner motivation. However, very few studies have explored the moderating effect of spiritual leadership on the effect of perceived respect on employee behavior (Chang et al., 2021).

To sum up, based on the broaden-and-build theory, this study uses striving at work and spiritual leadership to explore the influence of perceived respect on employees’ innovative behavior, as well as the internal mechanism of striving at work and the boundary condition of spiritual leadership. The theoretical contributions of this study are as follows. (1) This paper explores the stimulating effect of positive emotion on innovative behavior, broadening the research on the antecedents of innovative behavior. (2) Based on the broaden-and-build theory, this study reveals the internal dynamic mechanism through which thriving at work affects the relationship between perceived respect and innovative behavior. (3) This study examines the dual effects of spiritual stimulation, that is, the extrinsic stimulative and moderating effect of spiritual leadership, as well as the intrinsic psychological state of thriving at work. Moreover, this study extends the boundary conditions of the broaden-and-build theory.

## Hypothesis development

### Broaden-and-build theory

Broaden-and-build theory of positive emotions is an important theory to understand how positive emotions would facilitate favorable effects to individuals and organizations (Fredrickson, 2001). According to this theory, positive emotions expand the momentary thought-action repertoires and build personal resources, which will bring long-term adaptive values to individuals. According to this theory, positive emotions include joy, amusement, serenity, excitement, love, pride, interest, gratitude, hope, admiration, etc. (Fredrickson, 2013). The theory mainly includes two core assumptions: “the broaden hypothesis” and “the build hypothesis” (Fredrickson and Branigan, 2005). The broaden hypothesis holds that positive emotions would expand an individual’s attention, cognition, and action range, make an individual’s thinking mode more flexible, and expand an individual’s action tendency. The build hypothesis holds that positive emotions would construct individual lasting resources, including intellectual, physical, psychological, and social resources, to bringing long-term benefits such as personal growth and happiness. In this study, as perceived respect is defined as a positive emotional response to being recognized or appreciated, the broaden-and-build theory of positive emotions provides a theoretical basis for the empirical study. The broaden hypothesis is applicable to explain the main effect of perceived respect on employees’ innovative behavior. And the build hypothesis is applicable to explain the mediating effect of thriving at work on the relationship between perceived respect and employees’ innovative behavior. In addition, this study also explores the theoretical boundaries of broaden-and-build theory.

### Perceived respect and innovative behavior

Employees’ innovative behavior refers to their generation, promotion, and practice of innovative and meaningful ideas, products, processes, services, or methods in their work (Scott and Bruce, 1994). This behavior is not included in the regulations of the organization but generated by employees at the individual level. Innovative behavior is considered essential and conducive to the organization’s survival, development, and sustained advantage in today’s competitive global market (Campo et al., 2014).

Fredrickson points out that positive emotion is a good and pleasant feeling generated when an individual is affirmed by the outside world (Fredrickson, 2001). Perceived respect is defined as perceived worth accorded to one person by one or more others (Spears et al., 2006; Rogers and Ashforth, 2017). Appreciation for the individual’s characteristics and the recognition that an individual belongs to and is valued by an organization contribute to perceived respect (Grover, 2014), a positive emotion in which an individual has a sense of worth (e.g., recognition and affirmation). Scholars have noted that respect in the workplace contributes to employees’ performance and work effort (Vinarski-Peretz et al., 2011; Grover, 2021).

Given contextual factors in China such as the reform and opening-up policy from 1978 and the one-child policy from 1980, the new generation of employees have been raised in better material and economic conditions but are facing a more challenging and dynamic social environment (Zhu et al., 2011). Compared with older employees, the new generation of employees is more eager to express their opinions, shows more intention to change the world, and shows more distinct personalities, such as being independent, self-centered, and free (Zhou, 2007). They care more about personal feelings in the workplace and pay attention to democratic and respectful relationship with co-workers and the leader (Ren et al., 2018). Employees’ perception of respect enables them to experience their own value, pursue higher needs, exert their potential, and guide themselves to carry out more innovative behavior. Moreover, perceived respect from the organization could protect and develop the new-generation employees’ uniqueness and personalized resources, which are conducive to innovative behavior (Vinarski-Peretz et al., 2011).

According to the broaden-and-build theory, positive emotions (e.g., contentment, inspiration, and gratitude) can broaden a person’s momentary thought–action repertories (e.g., their scope of attention, cognition, and action; Fredrickson, 2001). In the workplace, people who are treated respectfully are more likely to feel a positive sense of self-worth (Grover, 2014). Perceived respect from their colleagues and leaders also gives employees a positive feeling that their contributions to the organization are recognized and valued (Ellemers et al., 2013). In this way, employees’ perception of respect broadens their thought–action tendencies such as exploring, taking in new information, and creating (Fredrickson, 2001), thus stimulating their innovative behavior. In addition, perceived respect in the workplace indicates that an employee deserves to be a member of a group and has equal rights to learn in the group (Honneth, 2004), which could enhance the employee’s positive association with their colleagues (Abid et al., 2018) and allow them to accumulate more resources from the organization for innovation.

Based on the above discussion, this study proposes the following:

*H1*: Perceived respect has a positive influence on employees’ innovative behavior.

### The mediating role of thriving at work

Thriving at work refers to the psychological state in which individuals experience the joint sense of vitality and learning or getting better at work (Spreitzer et al., 2005). The sense of vitality refers to the employees’ subjective experience of energy (Peterson and Seligman, 2004), whereas learning refers to an acquired mental state in which employees can apply knowledge or skills to their job to build their confidence and competence (Elliott and Dweck, 1988). Both aspects are critical to the outcome of thriving (Ali et al., 2018). Employees who lack vitality but still insist on learning might feel tired or even exhausted. Conversely, those who are motivated to work but lack opportunities to learn and grow are likely to stagnate (Mortier et al., 2016). Studies have demonstrated the crucial role of employees’ thriving at work in promoting the effectiveness and long-term development of both the employees and the organization (Porath et al., 2012). Thriving individuals are aware of their own potential and can observe how they and their behavior improve (Brown et al., 2017). Thriving employees’ vitality and energy can also directly influence organizational behavior and effectiveness (Carmeli and Spreitzer, 2009).

Thriving at work, which provides employees with powerful psychological energy, can positively influence their intrinsic motivation and drive them to perform innovative behavior (Anderson et al., 2014). In the workplace, employees’ innovative behavior consists of three main parts (Scott and Bruce, 1994), namely, recognizing a problem and coming up with new solutions or ideas, seeking ways to promote the solutions or ideas and getting support from colleagues, and making the solutions or ideas concrete and improving their applicability in the organization (Kanter, 1988). Thriving at work can have a positive effect on all three parts. First, a sense of thriving can endow employees with the vitality necessary for work (Anderson et al., 2014), which can, in turn, make them more likely to take the initiative to explore new technologies, ideas, and processes, facilitating their engagement in innovative behavior (Riaz et al., 2018; Liu et al., 2020). Second, employees who thrive at work can improve their professional abilities through a positive learning experience, which can provide them with favorable conditions to discover problems and find new solutions (Liu et al., 2020). When learning and growing, employees are more likely to acquire new expertise, which is conducive to the generation and implementation of novel ideas (Carmeli and Spreitzer, 2009). Finally, the new expertise acquired by employees can help them to make existing solutions more concrete and universally applicable, and can also boost employees’ confidence to change the status quo and create at work, thus facilitating their innovative behavior (Liu et al., 2020).

Therefore, this study proposes the following:

*H2*: Thriving at work has a positive effect on employees’ innovative behavior.

According to the broaden-and-build theory, perceived respect can broaden the scope of employees’ attention so that employees can keep an open mind to new ideas and activities. Perceived respect can also improve employees’ vitality and initiative, promoting their cognitive expansion and spontaneous learning (Fredrickson, 2001). Therefore, perceived respect could influence thriving at work. Positive emotions can help employees to become more psychologically adaptive to the working situation (Fredrickson, 2004). As a stimulus of positive emotions in the workplace, perceived respect can enhance employees’ adaptability to the changeable and competitive working environment, reducing the negative influence of the working situation (Spreitzer et al., 2005). In this way, employees can gain more autonomy and competence to thrive. Moreover, self-determination theory notes that satisfying employees’ basic psychological needs can enhance employees’ energy and encourage them to show enthusiasm and vitality (Deci and Ryan, 2008). As discussed earlier, perceived respect refers to a feeling of appraisal and recognition from the organization, which not only can satisfy employees’ basic psychological needs but also can be conducive to positive interaction and collaboration among colleagues (Abid et al., 2018). Satisfying employees’ need to be respected can make them more optimistic and confident. When employees feel valued and capable, they are more likely to keep learning and exploring, leading to increased vitality and learning (Li, 2018). A high level of trust and respect in interpersonal interactions could be a powerful force to enhance employees’ thriving at work (Carmeli and Spreitzer, 2009).

Therefore, the following hypothesis is proposed:

*H3*: Perceived respect has a positive effect on thriving at work.

Perceived respect, as a significant positive emotion, can help promote employees’ sense of thriving and then influence their innovative behavior and performance at work. More concretely, perceived respect can satisfy employees’ basic psychological needs, improve their feeling of self-esteem, and enhance their adaptability to the working environment, driving them to thrive (Ng, 2016). Simultaneously, thriving promotes employees’ initiative to engage in innovation. Thriving at work can broaden employees’ thought–action repertoires through the role of positive emotions, which can promote their innovative thinking and inner motivation to solve problems in the workplace (Hirt et al., 1997). Thriving can also help employees build their resources (e.g., psychological, intellectual, and social resources), which can contribute to their behavioral tendencies to innovation (Liu et al., 2020). To sum up, perceived respect affects employees’ innovative behavior through the effect of thriving at work.

Therefore, this study proposes the following:

*H4*: Thriving at work mediates the relationship between perceived respect and employees’ innovative behavior.

### The moderating role of spiritual leadership

Spiritual leadership, as an emerging paradigm in the broader research field of workplace spirituality, refers to a leadership style that stimulates and satisfies followers’ spiritual existence and spiritual needs (e.g., calling and membership) through intrinsic motivation (Fry, 2003). Studies have demonstrated that spiritual leadership can meet followers’ spiritual needs by transmitting vision, faith, and altruistic love to them, which could influence their attitudes and behaviors (Fry and Slocum, 2008; Fry and Kriger, 2009; Zou et al., 2020; Ali et al., 2022). People strive to be respected and valued in social relationships (Bergsieker et al., 2010), and most employees expect to be treated with respect in the workplace (Ng, 2016). Employees’ perception of respect is not derived from their own recognition and evaluations but based on other members’ evaluations and treatment within the organization (De Cremer, 2002). Particularly, the new generation of employees prefer democratic and equal interaction with others and desire recognition and respect from their leaders (Weyland, 2011). Therefore, the new generation of employees would perceive that they are being respected when their leaders and co-workers treat them in a normatively appropriate, supportive, and inclusive way (Bartel et al., 2012). It can reasonably be inferred that leadership style is an important factor affecting employees’ perceived respect, and a considerate and supportive leader can help employees to remain energized, work effectively, and achieve self-development (Abid et al., 2018).

Employees’ perceived respect can increase employees’ thriving at work, but this positive effect is moderated by spiritual leadership. First, Spiritual leadership can enhance employees’ perception of respect. By setting and publicizing the common organizational vision and goals, spiritual leader can improve organizational cohesion, give employees a sense of belonging to organizational, and make them feel that they are instrumental to the organization’s development (Fry&Kriger, 2009; Zou et al., 2020), thereby enhancing employees’ sense of being respected. Through affirming employees’ existing contributions, spiritual leadership endows employees with the confidence and faith to pursue goals, making them perceive more recognition and attention, and thus more respect, from the organization and the leader (Fry et al., 2011); Spiritual leadership can also meet employees’ need for psychological and social resources through expressing altruistic love, which also enhances their perceived respect (Fry and Slocum, 2008). Therefore, the stronger the role played by spiritual leadership, the behaviors, actions, and thoughts of employees can be recognized, tolerated, and encouraged to a greater extent, so as to improve their perceived respect, arouse more positive emotions, inject more vitality into employees, increase the internal motivation for learning, and then work more efficiently.

Second, spiritual leadership is a typical organizational situational factor that can enhance a sense of thriving among employees (Spreitzer et al., 2005). When spiritual leadership has a strong effect, employees are more likely to pursue higher level needs due to their spiritual survival needs being activated, which also awakens employees’ intrinsic motivation for self-enhancement (Taormina and Gao,2013). In order to meet higher level needs, employees become more energetic and proactive (Yang et al., 2020), which can give them the confidence to acquire and utilize new skills and develop their competence and confidence (Liu et al., 2020), leading to enhanced enthusiasm and vitality at work and, finally, thriving employees. In summary, when the effect of spiritual leadership is strong, the positive effect of employees’ perceived respect on thriving at work will be amplified. Contrastingly, when the level of spiritual leadership is low, because the new generation of employees attach importance to the realization of spiritual values, they lack the important organizational source of perceived respect (Fry, 2003), and also reduce the perception of respect and the understanding of the meaning of work, which leads to insufficient internal motivation of thriving at work.

Following these arguments, this study proposes the hypothesis:

*H5*: Spiritual leadership positively moderates the relationship between perceived respect and thriving at work, such that the positive effect of perceived respect on thriving at work is enhanced when the effect of spiritual leadership is strong.

Spiritual leadership can strengthen the mediating effect of thriving at work in the relationship between perceived respect and innovative behavior. In other words, when the effect of spiritual leadership is strong, employees are more likely to feel respected, resulting in increased thriving at work and more innovative behaviors. According to the broaden-and-build theory, employees with positive emotions are enthusiastic and able to follow innovative and unique thoughts, actions, and activities (Fredrickson, 2001). Under the influence of spiritual leadership, employees gain confidence and show increased initiative and enthusiasm because their spiritual need to be respected is satisfied, which is conducive to innovative behaviors (Zhang and Yang, 2020). The process of innovation is full of challenges and risks such as the characteristics of fuzzy innovation paths and changeable innovation benefits, which can increase employees’ job insecurity and the mental pressure to innovate (Bessant, 2003). Spiritual leadership helps form a trust-based and friendly working environment with a culture of mutual respect, within which employees have increased vitality, are more involved with their jobs and thrive (Fry and Slocum, 2008). Employees who thrive at work have greater psychological and physical advantages than those who do not (Tummers et al., 2018), which, in turn, enables them to take risks and innovate without worrying about potential mistakes and failure (Fry and Slocum, 2008). In addition, a spiritual work environment motivates employees’ innovative behaviors through improving their self-confidence, personal awareness, and intuition about their innovative ability (Fawcett et al., 2008; Pawar, 2009).

Therefore, this study proposes the following:

*H6*: Spiritual leadership positively moderates the mediating effect of thriving at work on the relationship between perceived respect and employees’ innovative behavior; that is, the mediating effect of thriving at work is enhanced when the effect of spiritual leadership is strong.

**Figure 1:**
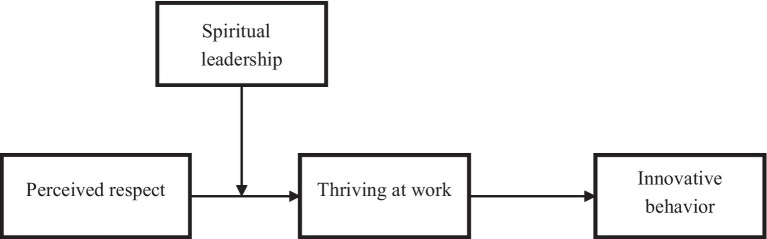
Theoretical model.

Based on the above, the theoretical model is shown in Figure 1.

## Materials and methods

### Participants and procedures

In this study, employees born after 1980 and their direct superiors from more than 10 enterprises of China in Beijing, Shenzhen, Guangzhou, Shanghai, and Xi‘an were selected as the research participants. The samples were mainly collected from the service industry, manufacturing industry, communication and IT technology, finance industry, and high-tech industry. The posts include ordinary staff, middle and senior professional technical posts, and management posts. The geographical industrial and position sources of samples were relatively diverse, which ensured external validity to a large extent. In order to avoid the homology error, paired leader–employee questionnaire was adopted to collect data. The questionnaires involved employee questionnaires and direct leader questionnaires. Employees answered questions related to their personal information, perceived respect, thriving at work, and perception of spiritual leadership, while the employees’ direct leaders evaluated the employees’ innovative behavior.

Before issuing the questionnaire, the research group communicated with the human resources management department of each enterprise. With the help of the human resource manager, we obtained an employee roster. We numbered the leaders and their direct subordinates according to the roster, and filled in the employee’s name on the corresponding leadership questionnaire, so as to facilitate the leaders to evaluate one by one. Each leader evaluated his four or five subordinates. The questionnaires delivered to the leaders and their corresponding employees in the form of independent paper envelopes, and the corresponding numbers are marked on the upper left corner of each envelope. For example, the envelope number of the No. 1 leader’s questionnaire was 1, and the envelope numbers of its four subordinates were 1–1, 1–2, 1–3, and 1–4. Under the guidance of the research group, some leaders and employees completed the questionnaires on site and returned them. Some questionnaires were brought back by leaders and employees to fill in separately, and then sealed and sent back. A total of 598 paired questionnaires were collected, 506 of which were valid after excluding unqualified questionnaires such as incomplete questionnaires or invalid answers, showing an effective response rate of 84.62%.

Male participants accounted for 53.56% of the employees, and female participants accounted for 46.44%. Most of the employees were aged younger than 25 years (27.3%), between 25 and 30 years (41.3%), or between 31 and 40 years (31.4%). The employees’ educational backgrounds consisted of less than junior college (7.4%), junior college (24.3%), a bachelor’s degree (58.8%), and a master’s degree or above (9.5%). In addition, 44.5% of the employees were married, while 54% were single or divorced. The employees had work tenure of less than 1 year (19.6%), 1–3 years (42.3%), 4–6 years (22.9%), 7–10 years (8.3%), and 11 years or more (6.9%). They worked for various enterprise types, namely, state-owned enterprises (25.1%), private enterprises (60.9%), foreign-funded enterprises (8.9%), and others (5.1%).

### Measuring

The variables involved in this study were perceived respect, innovative behavior, thriving at work, and spiritual leadership, which were measured using mature scales with good reliability and validity. We invited a professor, a post-doctoral fellow, and two bilingual doctoral students to participate in the “translator-translation” work. Considering cultural matching and ease of understanding, the English scale was translated into Chinese and then back into English. The Chinese scale was pre-filled in a small range, and finally, the research scale was determined. All items were assessed on a 7-point Likert scale ranging from 1 (completely disagree) to 7 (completely agree).

#### Perceived respect

*Perceived respect* was measured using the six-item scale developed by Blader and Tyler (2009). Example items are “My leader has great respect for my work,” “My leader respects my ideas about work,” and “My leader thanks me for my unique contribution to work.” In this study, Cronbach’s α for this scale was 0.930.

#### Innovative behavior

*Innovative behavior* was measured using the six-item scale developed by Scott and Bruce (1994). Example items are “My employee is always looking to apply new processes, techniques and method,” “My employee often comes up with innovative ideas,” and “My employee always finds a way to get the resources he/she needs to implement new ideas.” In this study, Cronbach’s α for this scale was 0.893.

#### Thriving at work

*Thriving at work* was measured using the scale developed by Spreitzer et al. (2005), which is a two-dimensional scale with eight items. Example items are “I am always full of energy at work,” “At work, I always take the initiative to learn,” and “I feel I am making progress in my work.” In this study, Cronbach’s α for this scale was 0.913.

#### Spiritual leadership

Spiritual leadership was assessed using the 17-item scale developed by Fry et al. (2005). Example items are “My leader describes the organizational vision to employees” and “My leader really cares about and values employees.” In this study, Cronbach’s α for this scale was 0.919.

#### Control variables

To prevent the influence of demographic variables, gender, age, marital status, educational background, working tenure, and enterprise type were selected as control variables in this study.

## Results

### Common method bias test

Harman’s single-factor test was used in this study to test for common method bias. The total variance explained was 68.89%, and the first factor explained 23.04% of the variance, which was less than 40% of the total variation, indicating that there was no serious common method bias in this study.

### Confirmatory factor analysis

To test the discriminant validity among perceived respect, innovative behavior, thriving at work, and spiritual leadership, Mplus 7.0 was used for confirmatory factor analysis. Table 1 presents the analysis results. Compared with the 1-, 2-, and 3-factor models, the 4-factor model had the best fitting indices (χ^2^/df = 2.981 < 3; TLI = 0.912 > 0.9; CFI = 0.918 > 0.9; RMSEA = 0.063 < 0.1; SRMR = 0.050), indicating that the four variables selected in this study had good discriminant validity.

**Table 1:** Confirmatory factor analysis results.

Model	χ2/df	TLI	CFI	RMSEA	SRMR
1-factor model(PR + TW + SL + IB)	7.930	0.691	0.711	0.117	0.101
2-factor model(PR + TW + SL, IB)	5.872	0.783	0.797	0.098	0.075
3-factor model(PR + TW, SL, IB)	4.727	0.834	0.845	0.086	0 0.092
4-factor model(PR, TW, SL, IB)	2.981	0.912	0.918	0.063	0.050

PR, Perceived Respect; IB, Innovative Behavior; TW, Thriving at Work; and SL, Spiritual Leadership.

### Descriptive statistics and correlations

The means, standard deviations, and correlation coefficients among perceived respect, innovative behavior, thriving at work, spiritual leadership, and control variables, as well as the Cronbach’s α coefficients for these variables, are shown in Table 2. The table shows that the Cronbach’s α of each variable was above 0.7, indicating that all of the scales used in this study had good reliability. In addition, perceived respect was significantly positively correlated with innovative behavior (*r* = 0.333, *p* < 0.01) and thriving at work (*r* = 0.520, *p* < 0.01), which preliminarily verified the hypotheses proposed in this study.

**Table 2:** Descriptive statistics and correlation coefficient.

Variables	*M*	*SD*	1	2	3	4	5	6	7	8	9	10
1.Gender	1.480	0.567	1.000									
2.Age	2.200	0.892	0.008	1.000								
3.EB	2.710	0.787	0.078	0.141**	1.000							
4.MS	1.580	0.605	0.311**	−0.406**	0.200**	1.000						
5.WT	2.420	1.128	0.121**	0.535**	0.040	−0.185**	1.000					
6.EP	2.000	0.922	0.180**	0.085	−0.165**	0.190**	0.111*	1.000				
7.PR	6.407	0.665	0.025	−0.011	−0.044	−0.039	0.048	0.028	(0.930)			
8.IB	5.894	0.895	−0.012	0.040	−0.007	−0.121**	0.029	0.078	0.333**	(0.893)		
9.SL	6.010	0.815	−0.014	0.042	−0.067	−0.077	0.042	0.047	0.616**	0.350**	(0.913)	
10.TW	5.864	0.785	−0.163*	0.037	−0.091*	−0.070	0.023	0.039	0.520**	0.289**	0.715**	(0.919)

*p < 0.05;

** *p* < 0.01;

*** *p* < 0.001.

Cronbach’s alpha reliabilities are reported in the parentheses. EB, Education Background; MS, Marital Status; WT, Work Tenure; EP, Enterprise Property; PR, Perceived Respect; IB, Innovative Behavior; SL, Spiritual Leadership; and TW, Thriving at Work.

### Hypothesis testing

#### The main effect test of perceived respect and innovative behavior

The hierarchical regression analysis results are shown in Table 3. After controlling for the variables of gender, age, educational background, marital status, work tenure, and enterprise type, model 6 indicated that perceived respect had a significant positive effect on innovation behavior (*β* = 0. 326, *p* < 0.001), which supported hypothesis 1.

**Table 3:** Hierarchical regression analysis results.

Variable	Thriving at work				Innovative behavior			
	M1	M2	M3	M4	M5	M6	M7	M8
Gender	−0.172^***^	−0.190^***^	−0.176^***^	−0.176^***^	0.018	0.006	0.107	0.037
Age	0.036	0.076	0.045	0.043	−0.057	−0.032	−0.067	−0.044
ED	−0.077	−0.067	−0.046	−0.044	0.056^**^	0.062^**^	0.089	0.073^**^
MS	0.008	0.045	0.063	0.063	−0.183	−0.160	−0.274^**^	−0.167
WT	0.024	−0.012	−0.001	−0.005	0.007	−0.015	0.001	−0.013
WP	0.050	0.034	0.014	0.014	0.123^*^	0.113^*^	0.105^*^	0.107^*^
PR		0.524^***^	0.139^***^	0.200^***^		0.326^***^		0.241^***^
TW							0.334^***^	0.162^**^
SL			0.626^***^	0.633^***^				
PR*SL				0.118^**^				
R-sq	0.038	0.310			0.029	0.135	0.111	0.153
Adjusted R-sq	0.027	0.300			0.017	0.122	0.099	0.139
ΔR-sq	0.038	0.272			0.029	0.105	0.082	0.018
F	3.310^**^	31.932^***^			2.491^*^	11.063^***^	8.917^***^	11.203^***^

EB, Education Background; MS, Marital Status; WT, Work Tenure; EP, Enterprise Property; TW, Thriving at Work; and PR*SL, Perceived Respect*Spiritual Leadership.

* *p* < 0.05;

** *p* < 0.01;

*** *p* < 0.001.

#### The mediating effect test of thriving at work

Model 2 in Table 3 shows that perceived respect had a significant positive effect on thriving at work (*β* = 0.524, *p* < 0.001), and model 7 showed that thriving at work had a significant positive effect on innovative behavior (*β* = 0.334, *p* < 0.001), supporting hypotheses 2 and 3. After adding the data for perceived respect and thriving at work simultaneously, model 8 showed that the effect of perceived respect on innovative behavior was significantly weakened (*β* = 0.241, *p* < 0.001), while the effect of thriving at work on innovative behavior remained significant (*β* = 0.162, *p* < 0.01), indicating that thriving at work partly mediated the relationship between perceived respect and innovative behavior. A bootstrapping sampling method was carried out with 5,000 replicates to further verify the mediating effect of thriving at work. The results showed that the 95% CI was [0.065, 0.295], excluding zero, indicating that thriving at work played a significant mediating role between perceived respect and innovative behavior. Thus, hypothesis 4 was verified.

#### The moderating effect of spiritual leadership

**Figure 2:**
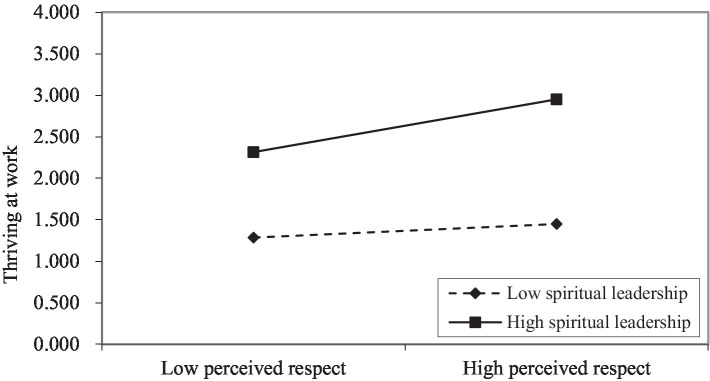
The moderating effect of spiritual leadership.

Model 4 in Table 3 indicated that the interaction item of perceived respect and spiritual leadership (*β* = 0. 118, *p*. < 001) was significant, showing that spiritual leadership played a moderating role in the relationship between perceived respect and thriving at work. To describe the moderating effect of spiritual leadership more clearly, a figure of a simple slope moderating effect (±1 *SD*) was provided. As shown in Figure 2, perceived respect had a more significant positive effect on thriving at work when the effect of spiritual leadership was strong, supporting hypothesis 5.

Finally, the moderated mediating effect was tested using the PROCESS bootstrap method. As shown in Table 4, when the effect of spiritual leadership was weak (TTU = M^−1^ SD), the mediating effect of thriving at work was not significant (*β* = 0.021, 95% CI [−0.005, 0.051], including zero). However, when the effect of spiritual leadership was strong (TTU = M + 1 SD), the mediating effect of thriving at work was significant (*β* = 0.060, 95% CI [0.013, 0.118], excluding zero). Moreover, the mediating effect of thriving at work was significantly different between low (TTU = M^−1^ SD) and high (TTU = M + 1 SD) spiritual leadership (*β* = 0. 038, 95% CI [0.003, 0.086], excluding zero), indicating that spiritual leadership had a moderating effect in the mediating relationship between perceived respect, thriving at work and innovative behavior. Hypothesis 6 was thus supported.

**Table 4:** The moderated mediating effect testing results.

Effect	*B*	SE	95% CI	
			LLCI	ULCI
Low SL	0.021	0.014	−0.005	0.051
High SL	0.060	0.027	0.013	0.118
Difference	0.038	0.021	0.003	0.086

## Discussion

This study elucidates how and when the respect perceived by the new generation of employees affects their innovative behavior from the perspective of the broaden-and-build theory. The new generation of employees has gradually become the main force to promote social and economic development (Ren et al., 2018). They are more educated, independent, and self-centered, pay more attention to personal feelings and experience at work than older employees, and have a strong desire to make a difference in the workplace (Zhu et al., 2015). This study explored the effect of perceived respect on innovative behavior, as well as the mediating effect of thriving at work and the moderating function of spiritual leadership. The results showed that perceived respect promoted employees’ innovative behavior both directly and indirectly through the mediating effect of thriving at work. In addition, spiritual leadership performed a significant moderating function in the relationship between perceived respect, thriving at work, and innovative behavior. The hypotheses results are summarized in Table 5. The results of this study provide theoretical implications and practical guidance for the innovative and sustainable development of both organizations and society.

**Table 5:** Hypothesis test results.

Hypotheses		Findings
H1	Perceived respect has a positive influence on employees’ innovative behavior.	Supported
H2	Thriving at work has a positive effect on employees’ innovative behavior.	Supported
H3	Perceived respect has a positive effect on thriving at work.	Supported
H4	Thriving at work mediates the relationship between perceived respect and employees’ innovative behavior.	Supported
H5	Spiritual leadership positively moderates the relationship between perceived respect and thriving at work	Supported
H6	Spiritual leadership positively moderates the mediating effect of thriving at work on the relationship between perceived respect and employees’ innovative behavior.	Supported

### Theoretical contributions

First, based on the broaden-and-build theory, this study revealed the internal dynamic mechanism of thriving at work in the relationship between perceived respect and innovative behavior, which deepened the initiative and sustainability of employees’ innovative behavior. Innovation is accompanied by numerous obstacles and setbacks that take employees out of their comfort zone (Staw, 1995). In the face of the increasingly complex and competitive organizational environment and the challenges brought by innovative activities, individuals need not only the vitality to innovate but also the ability to constantly learn and grow (Spreitzer et al., 2005). Employees who thrive at work have the enthusiasm to do the current work well and seek long-term development (Porath et al., 2012). Furthermore, they can constantly optimize work procedures and put forward improvement plans and constructive opinions that are conducive to the innovative development of the organization (Liu et al., 2020). The few studies on the relationship between respect and innovation have mostly focused on cognitive mechanisms such as self-efficacy (Ng, 2016) and relation information processing (Carmeli et al., 2015). Thriving at work highlights the employees’ internal driving force and personal will. Its impact on innovation behavior is stronger and more lasting than external incentives, and it is in line with the requirements of innovation.

Second, this study investigated the dual spiritual stimulation effect of perceived respect on innovation behavior, namely, the extrinsic motivation and moderating effect of spiritual leadership, as well as the internal psychological state and mediating effect of thriving at work. To date, scholars have mainly explored the influence and affecting mechanism of spiritual leadership on employees’ behavior (Chen et al., 2019; Anser et al., 2021; Ali et al., 2022). Although studies have confirmed the moderating effect of some leadership styles such as transformational leadership (Frieder et al., 2018; Meng et al., 2020), little attention has been paid to the moderating effect of spiritual leadership. Our results showed that spiritual leadership is an important situational source of employees’ perceived respect, which can strengthen the effect of the positive emotion of perceived respect on employees’ innovative behavior through thriving at work, expanding the understanding of spiritual leadership. Leaders are often the core figures in an organization. Their personal charisma can indirectly or directly influence employees’ attitudes, behavior, and performance (Abid et al., 2018). When the effect of spiritual leadership is strong, employees sense that the organization and leader encourage, respect, and support them to innovate (Fry, 2003). Such employees are more willing to learn new knowledge and skills, share resources and ideas with colleagues, and help each other overcome difficulties, thus improving their innovative behavior and the innovative development of the organization (Fry and Slocum, 2008; Pawar, 2009). Thus, the results of this study enrich and expand the explanation scope and effect mechanism of both spiritual leadership and thriving at work. In addition, limited literature to date has explored the boundary conditions of the broaden-and-build theory. Accordingly, there is insufficient theoretical discussion and empirical research on the circumstances under which positive emotions are most likely to promote employees’ positive attitudes and behaviors (Vacharkulksemsuk and Fredrickson, 2013). This study has introduced spiritual leadership as the moderator in the relationship between perceived respect, thriving at work, and innovative behavior, responding to the call to find the theoretical boundaries of the broaden-and-build theory (Vacharkulksemsuk and Fredrickson, 2013; Waters, 2020).

Finally, this paper explores the stimulating effect of perceived respect on innovative behavior, which broadens the research on the antecedents of innovative behavior. We hold the opinion that perceived respect is an important positive emotion that employees receive from colleagues and leaders in the workplace (Abid et al., 2018), and is also one of the basic psychological needs employees have at work (Taormina and Gao, 2013). Employees who perceive that they are respected in the workplace gain a sense of membership, recognition, and psychological safety; therefore, they show increased loyalty and a sense of responsibility toward the organization (Grover, 2014). They are more willing to work harder and pay more attention to their long-term development, showing more positive working behavior and seeking higher targets (e.g., generating new ideas, acquiring new skills, and turning those ideas into reality) (Vinarski-Peretz et al., 2011; Abid et al., 2018) than those employees who do not feel respected. Research on the antecedents of individual innovation has mostly focused on perspectives such as (Baer, 2010; Liang et al., 2022), problem-solving competencies (Gu et al., 2015), and organizational culture and supportive investment (Lin, 2011). The intrinsic driving effect of positive emotions in promoting employees’ innovative behavior and the strong demand of innovation for positive emotions has not received much attention. Our findings are consistent with previous studies on the impact of respect on employees’ positive behavior (Abid et al., 2018; Friedman et al., 2018; Ng et al., 2021). Scholars often regard perceived respect as an external situational or informational factor (Carmeli et al., 2015). However, we believe that perceived respect is an emotional result, and that employees who feel respected and recognized are more likely than others to decide and be motivated to exhibit positive behavior. Therefore, we enrich the research on the affective antecedents of employees’ innovative behavior.

### Practical implications

First, our study confirmed that perceived respect is positively correlated with innovative behavior. The new generation of employees, in particular, care about feeling respected and recognized at work (Weyland, 2011); therefore, managers should pay attention to constructing an organizational culture of mutual help and respect, providing a good working atmosphere for employees (Abid et al., 2018). Only when employees perceive respect from the working environment can they engage in higher occupational pursuits and produce a more positive attitude and behavior conducive to innovation. Such employees would have the confidence to complete their goals, be willing to share resources and information with colleagues, and seek innovative breakthroughs.

Second, the results of this research showed that thriving at work is positively related with innovative behavior. In other words, employees who thrive at work are more energetic, good at learning and acquiring resources, and more eager to produce innovative behavior than others. Therefore, managers should pay attention to activate employees’ thriving at work, thus improving their initiative to innovate and the sustainability of their innovation (Boyd, 2015). Organizations could take thriving at work as one of the standards to be considered when selecting employees and assign thriving employees to positions that require high levels of innovative ability. Organizations could also provide more training and learning resources for employees to strengthen their knowledge and understanding of innovation, making them realize the importance of innovation as well as the significance of innovation to enterprises and society (Niessen et al., 2012).

Finally, our results showed that spiritual leadership positively moderates the relationship between perceived respect and thriving at work as well as the mediating effect of thriving at work between perceived respect and innovative behavior. That is, spiritual leadership plays a role in explaining the beneficial effects of perceived respect among employees. As a leader, it is necessary to stimulate employees’ internal motivation. Managers could set an example for employees to learn, pay attention to employees’ emotional state, actively affirm their contributions, meet their spiritual needs, and give them care, respect, and support (Hunsaker, 2020). Managers should also protect employees’ unique characteristics, give them opportunities to try new ways of working, and encourage them to put forward different opinions (Zhang and Yang, 2020). In this way, employees would be not only able but also more willing to innovate, thus proactively promoting their innovative behavior.

### Limitations and directions for future research

This study has some limitations that need to be improved on in future research. First, the emotion promotion mechanisms of innovation need to be studied further. Based on the broaden-and-build theory, this paper supports the notion that perceived respect as a positive emotion can effectively improve employees’ innovative behavior. However, the organizational context is dynamic and complex, with various intertwined situational cues，such as different types of leadership styles present in the organization. Accordingly, employees’ perceptions of emotions may vary. Thus, the influence of multiple emotional factors on employees’ innovative behavior deserves further discussion.

Second, this study explored a moderated mediation model of perceived respect and innovative behavior by introducing the broaden-and-build theory. However, this theoretical perspective mainly focuses on exploring positive emotions and has a limited effect on the influencing mechanism of other types of factors. Future research could explore more influencing factors from other theoretical perspectives to improve and enrich the theoretical development of the field of organizational innovation.

Finally, in terms of data collection, this study used a leader–employee paired sample to collect data, an approach that can reduce homologous variance. The data analysis results of this study showed that there was no serious common method bias, and the variables had good reliability and discriminant validity. However, because it was difficult to obtain data from multiple time points, we conducted cross-sectional research. Perhaps because of the cross-sectional data, this study has a high correlation (the correlation between spiritual leadership and thriving at Work). In the future, we could collect data at multiple time points or conduct dynamic research in combination with longitudinal cases to reveal the causal relationship between the variables more accurately and vividly.

## Data availability statement

The original contributions presented in the study are included in the article/supplementary material, further inquiries can be directed to the corresponding authors.

## Author contributions

LZ: conceptualization, software, investigation, methodology, data curation, formal analysis, and writing—original draft. PL: conceptualization, investigation, resources, writing—reviewing, supervision, and project administration. FZ: writing—original draft, writing—reviewing, and editing. SX: interpretation of the results and writing—reviewing. YL: discussion of the results and investigation process. All authors contributed to the article and approved the submitted version.

## Funding

This work was supported by National Office for Philosophy and Social Sciences of China (20BGL136) and Doctor program of Nanyang Normal University (ZX2017024).

## Conflict of interest

The authors declare that the research was conducted in the absence of any commercial or financial relationships that could be construed as a potential conflict of interest.

## Publisher’s note

All claims expressed in this article are solely those of the authors and do not necessarily represent those of their affiliated organizations, or those of the publisher, the editors and the reviewers. Any product that may be evaluated in this article, or claim that may be made by its manufacturer, is not guaranteed or endorsed by the publisher.
